# Cannabidiol rather than *Cannabis sativa* extracts inhibit cell growth and induce apoptosis in cervical cancer cells

**DOI:** 10.1186/s12906-016-1280-0

**Published:** 2016-09-01

**Authors:** Sindiswa T. Lukhele, Lesetja R. Motadi

**Affiliations:** Department of Biochemistry, North-west University (Mafikeng campus), Private Bag X1290, Potchefstroom, 2520 South Africa

**Keywords:** Apoptosis, Cervical cancer, Cannabidiol, *Cannabis sativa*

## Abstract

**Background:**

Cervical cancer remains a global health related issue among females of Sub-Saharan Africa, with over half a million new cases reported each year. Different therapeutic regimens have been suggested in various regions of Africa, however, over a quarter of a million women die of cervical cancer, annually. This makes it the most lethal cancer amongst black women and calls for urgent therapeutic strategies. In this study we compare the anti-proliferative effects of crude extract of *Cannabis sativa* and its main compound cannabidiol on different cervical cancer cell lines.

**Methods:**

To achieve our aim, phytochemical screening, MTT assay, cell growth analysis, flow cytometry, morphology analysis, Western blot, caspase 3/7 assay, and ATP measurement assay were conducted.

**Results:**

Results obtained indicate that both cannabidiol and *Cannabis sativa* extracts were able to halt cell proliferation in all cell lines at varying concentrations. They further revealed that apoptosis was induced by cannabidiol as shown by increased subG0/G1 and apoptosis through annexin V. Apoptosis was confirmed by overexpression of p53, caspase 3 and bax. Apoptosis induction was further confirmed by morphological changes, an increase in Caspase 3/7 and a decrease in the ATP levels.

**Conclusions:**

In conclusion, these data suggest that cannabidiol rather than Cannabis sativa crude extracts prevent cell growth and induce cell death in cervical cancer cell lines.

## Background

*Cannabis sativa* is a dioecious plant that belongs to the *Cannabaceae* family and it originates from Central and Eastern Asia [11, 28]. It is widely distributed in countries including Morocco, South Africa, United States of America, Brazil, India, and parts of Europe [14, 28]. *Cannabis sativa* grows annually in tropical and warm regions around the world [11]. Different ethnic groups around the world use *Cannabis sativa* for smoking, preparing concoctions to treat diseases, and for various cultural purposes [17]. According to [28], it is composed of chemical constituents including cannabinoids, nitrogenous compounds, flavonoid glycosides, steroids, terpenes, hydrocarbons, non-cannabinoid phenols, vitamins, amino acids, proteins, sugars and other related compounds. Cannabinoids are a family of naturally occurring compounds highly abundant in *Cannabis sativa* plant [1, 6, 14, 24]. Screening of *Cannabis sativa* has led to isolation of at least 66 types of cannabinoid compounds [1, 14, 30]. These compounds are almost structurally similar or possess identical pharmacological activities and offer various potential applications including the ability to inhibit cell growth, proliferation and inflammation [22]. One such compound is cannabidiol (CBD), which is among the top three most widely studied compounds, following delta-9-tetrahydrocannabinol (Δ^9^-THC) [14]. It has been found to be effective against a variety of disorders including neurodegerative disorders, autoimmune diseases, and cancer [24, 25]. In a research study conducted by [26], it was found that CBD inhibited cell proliferation and induces apoptosis in a series of human breast cancer cell lines including MCF-10A, MDA-MB-231, MCF-7, SK-BR- 3, and ZR-7-1 and further studies found it to possess similar characteristics in PC-3 prostate cancer cell line [25]. However, to allow us to further our studies in clinical trials a range of cancers in vitro should be tested to give us a clear mechanism before we can proceed. *Cannabis sativa* in particular cannabidiol, we propose it plays important role in helping the body fight cancer through inhibition of pain and cell growth. Therefore, the aim of this study was to evaluate the cytotoxic and anti-proliferative properties of *Cannabis sativa* and its isolate, cannabidiol in cervical cancer cell lines.

## Methods

### Materials

An aggressive HeLa, a metastatic ME-180 and a primary SiHa cell lines were purchased from ATCC (USA, MD). Camptothecin was supplied by Calbiochem® and cannabidiol was purchased from Sigma-Aldrich and used as a standard reference.

#### Plant collection and preparation of extracts

Fresh leaves, stem and roots of *Cannabis sativa* were collected from Nhlazatshe 2, in Mpumalanga province. Air dried *C. sativa* plant material was powdered and soaked for 3 days in *n*-hexane, ethanol and *n*-butanol, separately. Extracts were filtered using Whatman filter paper and dried. Dimethyl sulfoxide was added to dried extracts to give a final concentration of a 100 mg/ml. Different concentrations (50, 100, and 150 μg/ml) of *C. sativa* extracts were prepared from the stock and used in treating cells during MTT assay. HPLC-Mass spectrophotometry was performed to verify the presence of cannabidiol in our extracts. The plant was identified by forensic specialist in a forensic laboratory in Pretoria. The laboratory number 201213/2009 and the voucher number is CAS239/02/2009.

#### Cell culture

HeLa, ME-180 and SiHa were cultured in Dulbecco’s Modified Eagle Media (DMEM) supplemented with 10 % Fetal Bovine Serum (FBS) (Highveld biological,) and 1 % penicillin/streptomycin (Sigma, USA). Cells were maintained at 37 °C under 5 % of carbon dioxide (CO_2)_ and 95 % relative humidity. After every third day of the week, old media was removed and replaced with fresh media, to promote growth until the cells reach a confluence of ~70–80 %.

### Methods

#### MTT assay

Ninety microlitres of HeLa and SiHa cells were seeded into 96-well plates at 5×10^3^ cells per well and incubated overnight at 37 °C under 5 % CO_2_ and 95 % relative humidity to promote cell attachment at the bottom of the plate. Media was changed and the cells were treated with *Cannabis sativa* plant extracts at various concentrations (0, 50, 100, and 150 μg/ml (*w*/*v*)) for 24 h. After 24 h, the cells were treated with 10 μl of (5 mg/ml) MTT reagent (3-[4, 5-dimethylthiazol-2-yl]-2, 5-diphenyltetrazolium bromide) for 4 h at 37 °C under 5 % CO_2_. Ninety microlitres of DMSO was added into each well including wells containing media only and serves as a blank, to dissolve formazan crystals. Camptothecin and DMSO were included as controls. Optical density was measured using a micro plate reader (Bio-Rad) at 570 nm to determine the percentage of viable cells and account for cell death induced according to the outlined equation below:

$$ \%\ \mathrm{Cell}\ \mathrm{viability}=\frac{\mathrm{Absorbance}\ \mathrm{of}\ \mathrm{treated}\ \mathrm{cells}-\mathrm{Absorbance}\ \mathrm{of}\ \mathrm{blank}}{\mathrm{Absorbance}\ \mathrm{of}\ \mathrm{untreated}\ \mathrm{cells}-\mathrm{Absorbance}\ \mathrm{of}\ \mathrm{blank}}\times 100 $$

#### Cell growth analysis

Before seeding cells, a 100 μl of media was added to the 16 well E-plate and placed in the incubator to record background readings. A blank with media only was included to rule the possibility of media having a negative effect on the cells. In each well of the E-plate, 1×10^4^ cells were seeded and left in the incubator for 30 min to allow the cells to adhere to the bottom of the plate. The E-plate was placed and locked in the RTCA machine and experiment allowed to run for 22 h prior to the addition of the test compounds. Cells were treated with various concentrations (0, 50, 100, 150 μg/ml) of *C. sativa* hexane extract. Following treatment, the experiment was allowed to run for a further 22 h. Camptothecin (0.3 μM (*v*/*v*)) and DMSO (0.1 % (*v*/*v*)) were used as controls for comparative purposes. Procedure was repeated for *C. sativa* butanol extract.

#### Apoptosis assay

Cells were washed twice with 100 μl of cold Biolegend’s cell staining buffer followed by resuspension in 100 μl of Annexin V binding buffer. A 100 μl of cell suspension was transferred into 15 ml falcon tube and 5 μl of FITC Annexin V and 10 μl of Propidium iodide solution (PI) were added into untreated and treated cell suspension. The cells were gently vortexed and incubated at room temperature (25 °C) in the dark for 15 min. After 15 min, 400 μl of Annexin V binding buffer was added to the cells. The stained cells were analysed using FACSCalibur (BD Biosciences, USA).

#### Morphological analysis

Five hundred microliter of 1×10^4^ cells was added onto a 6-well plate containing coverslips. The plate was incubated overnight to allow the cells to attach. Following attachment, media was removed and cells were washed twice with PBS, prior to incubation with IC_50_ of Cannabis sativa extracts for 24 h. After 24 h, media was removed and cells were washed twice with PBS. Four percent (4 %) was added into each well and the plate incubated for 20 min at room temperature, to allow efficient fixation of cells. Cells were washed twice with PBS and once with 0.1 % BSA wash buffer and further stained with DAPI and Annexin V/FITC for 5 min. BX-63 Olympus microscope (Germany) was used to visualize the cells.

#### Mitochondrial assay (ATP detection)

Twenty five microlitres of 1×10^4^ cells per well were plated in a white 96-well luminometer plate overnight. Cells were treated with 25 μl of IC50 concentrations of Cannabis sativa crude extracts and cannabidiol dissolved in a glucose free media supplemented with 10 mM galactose. The plate was incubated at 37° in a humidified and CO2-supplemented incubator for a period of 24 h. Fifty microlitres of ATP detection reagent was added to each well and the plate further incubated for 30 min. Luminescence was measured using GLOMAX (Promega, USA). The assay was conducted in duplicates and ATP levels were reported as a mean of Relative Light Units (RLU). The following formula was used to calculate the ATP levels in RLU:

$$ \mathbf{R}\mathbf{L}\mathbf{U}=\mathbf{Luminescence}\left(\mathbf{sample}\right)-\mathbf{Luminescence}\left(\mathbf{blank}\right) $$

#### Caspase 3/7 activity

A hundred microliters of 1×10^4^ cells were plated overnight on a 96-well luminometer plate and allowed to attach overnight. The next day, cells were treated with 0.3 μM camptothecin and the IC_50_ concentrations of *Cannabis sativa* crude extracts and further incubated for a period of 24 h. Caspase-Glo 3/7 assay was performed according to manufacturer’s protocol (Promega, USA). Briefly, following treatment, media was replaced with caspase glo 3/7 reagent mixed with a substrate at a ratio of 1:1 *v*/*v* of DMEM: Caspase-glo 3/7 reagent and was incubated for 2 h at 37 °C in 5 % CO_2._ Luminescence was quantified using GLOMAX from Promega (USA). The assay was conducted in duplicates and caspase 3/7 activity was reported as a mean of Relative Light Units (RLU). The following formula was used to calculate caspase 3/7 activity in RLU:

$$ \mathbf{R}\mathbf{L}\mathbf{U}=\mathbf{Luminiscence}\left(\mathbf{sample}\right)-\mathbf{Luminiscence}\left(\mathbf{blank}\right) $$

#### Cell cycle analysis

Cells were harvested with 2 ml of 0.05 % trypsin-EDTA. Ten millilitres of media was added to the cells to inactivate trypsin and the cell suspension was centrifuged at 1500 rpm for 10 min. The supernatant was discarded and pellet was re-suspended twice in 1 ml PBS. Cell suspension was centrifuged at 5000 rpm for 2–5 min and PBS was discarded. Seven hundred microlitres of pre-chilled absolute ethanol was added to the cell suspension followed by storage at −20 °C for 30 min, to allow efficient permeabilization and fixing of the cells. After 30 min, cells were centrifuged at 5000 rpm for 5 min to remove ethanol. The pellet was washed twice with PBS and centrifuged at 5000 rpm to remove PBS. Five hundred microlitres of FxCycle™ PI/RNase Staining solution (Life technologies, USA) was added to the cells and vortexed for 30 s (sec). The cells were analysed with FACSCalibur (BD Biosciences, USA).

#### Western blot

Following 24 h of treatment with IC_50_ concentrations, cells were lysed using RIPA buffer (50 mM Tris-HCl pH 7.4, 150 mM NaCl, 1 % NP-40, 0.1 % SDS, 2 mM EDTA). Protein content was measured by the BCA assay and equal amounts were electrophoresed in SDS polyacrylamide gel and then transferred onto nitrocellulose membranes. Membranes were subsequently immunoblotted with Anti-mouse monoclonal p53, Bcl-2, Bax, RBBP6, Caspase-3 and -9 antibodies were used at 1:500–1000 dilutions as primary antibodies, while a goat anti-mouse horseradish peroxidise-conjugated horse IgG (Santa Cruz, USA) were used at a 1:2000 dilution as a secondary antibody. The membranes were developed using Chemiluminescence detection kit (Santa Cruz Biotechnology, CA). The membranes were imaged using a Biorad ChemiDoc MP.

#### Data analysis

Experiments were performed in duplicates. Statistical analysis of the graphical data was expressed as the mean standard deviation. The *p*-value was analysed in comparison to the untreated using Students *t*-Test wherein *p* < 0.05 was considered as significant.

## Results

### Effect of *Cannabis sativa* and cannabidiol on SiHa, HeLa, and ME-180 cells

**Fig. 1:**
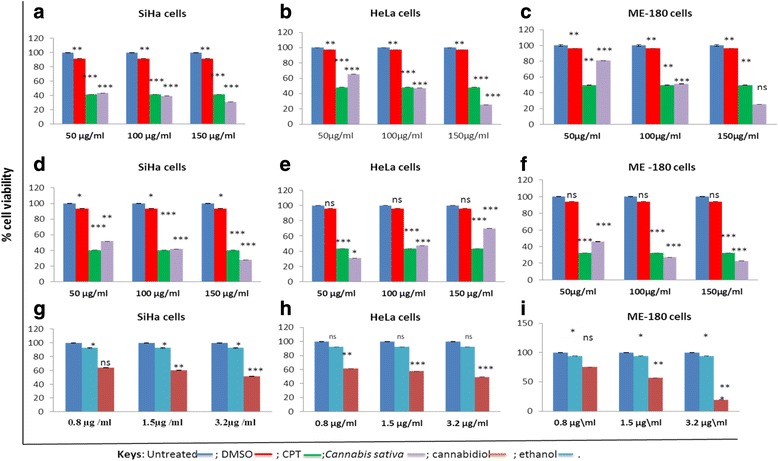
Representative cell viability bar graphs of cervical cancer cell lines. MTT assay was conducted to determine IC_50_ following incubation of SiHa, HeLa, and ME-180 cells with different doses of butanol extract (**a**, **b**, **c**), hexane extract (**d**, **e**, **f**), and cannabidiol extract (**g**, **h**, **i**) for a period of 24 h. Data was expressed as mean value ± standard deviation (SD). The level of significance was determined using Students *t*-Test with ^ns^representing *p* > 0.05, ***represents *p* < 0.001, **represents *p* < 0.01, and *represents *p* < 0.05

To determine half maximal inhibitory concentration (IC50) for both *Cannabis sativa* and cannabidiol, MTT assay was used. Camptothecin, as our positive control, significantly reduced cell viability in SiHa (40.36 %), HeLa (47.19 %), and ME-180 cells (32.25 %), respectively. As shown in Fig. 1a and d, the IC50 was cell type dependent rather than time dependent with SiHa at less than 50 μg/ml in both butanol (56 %) and hexane (48.9 %). Similarly IC_50_ in HeLa was at 100 μg/ml at *p* < 0.001 (Fig. 1b). while ME-180 cells treated with butanolic extract exhibited an IC_50_ of a 100 μg/ml, reducing viability to 48.6 % (Fig. 1c) and hexane extracts IC_50_ was at 50 μg/ml with 54 % death (Fig. 1f). This was not the case in cannabidiol with SiHa (51 %) and HeLa (50) IC_50_ at a much lower dose (3.2 μg\ml) while ME-180 cells (56 %) at 1.5 μg\ml when compared to *Cannabis sativa* extracts (*p* < 0.001) (Fig. 1g–i). Dimethyl sulfoxide (DMSO) was included as a vehicle control and it inhibited between 4 and 11 %. Whereas ethanol exhibited between 7.3 and 7.8 % since cannabidiol was alcohol extracted.

**Fig. 2:**
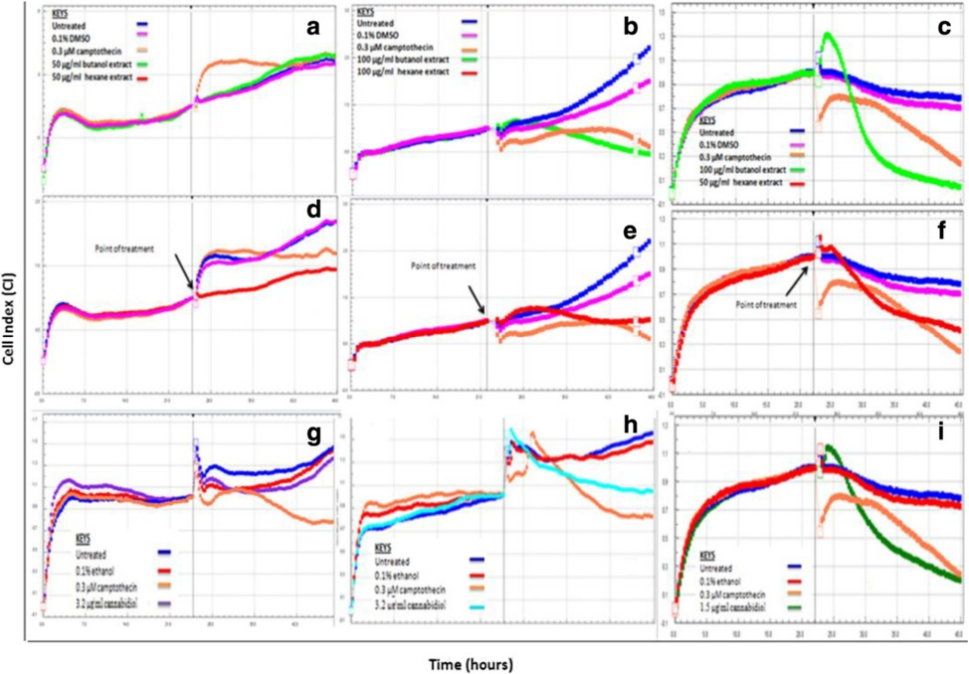
xCELLigence analysis of the cell growth pattern after treatment of cervical cancer cells with *Cannabis sativa* extracts and cannabidiol. SiHa (**a**, **d**, **g**), HeLa (**b**, **e**, **h**), and ME-180 (**c**, **f**, **i**) cells were seeded for a period of 22–24 h, followed by treatment with IC_50_ concentration of butanol (**a**, **b**, **c**), hexane (**d**, **e**, **f**), and cannabidiol (**g**, **h**, **i**)

### Effect of *Cannabis sativa* extracts and cannabidiol on cell growth of cervical cancer cells

**Fig. 3:**
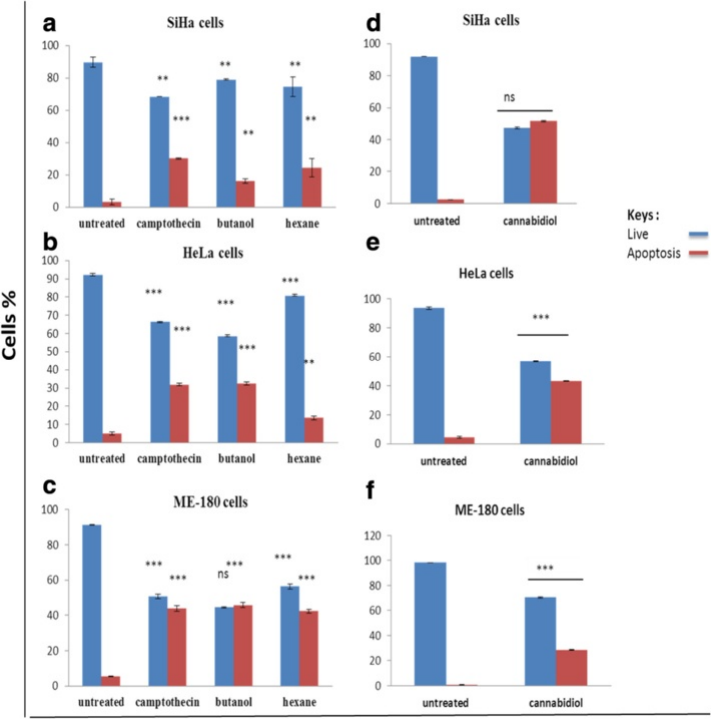
Apoptosis assessment following treatment of cervical cancer cells with IC_50_ concentrations of *Cannabis sativa* extract and cannabidiol. These bar graphs are a representative of apoptosis induction in SiHa (**a** and **d**), HeLa (**b** and **e**), and ME-180 (**c** and **f**) cells. Cells were treated with IC_50_ of *Cannabis sativa* extracts and cannabidiol for a period of 24 h and further stained with Annexin-V/PI. Data represented as mean ± standard deviation with ***p < 0.001, **p < 0.01 and ^ns^ *p* > 0.05 representing the level of significance in comparison to the untreated

The IC_50_ obtained during MTT assay was tested for their ability to alter cell viability in real time. An impedance based system was employed to evaluate the effect of *Cannabis sativa* and cannabidiol on SiHa, HeLa, and ME-180. Cells were seeded in an E-plate and allowed to attach. Cells were further treated with IC_50_ for a period of 22–24 h, depending on their doubling time. Continuous changes in the impedance were measured and displayed as cell index (CI). Little can be read from xCELLigence except that cannabidiol in all cell lines has shown to reduce cell index while the plant extract had mixed results sometimes showing reduction on the other hand remained unchanged (Fig. 3). Suggesting that cannabidiol is the most effective compound.

**Fig. 4:**
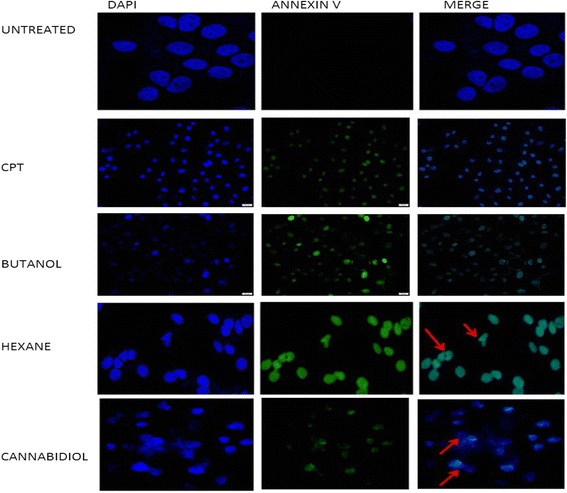
Morphological analysis and assessment of apoptosis in SiHa cells stained with DAPI and Annexin V dye. Cells were incubated with IC_50_ of *Cannabis sativa* extracts for a period of 24 h. Cells were stained with Annexin V and counterstained with DAPI. BX63-fluorescence confocal microscopy was used to visualize the cells

**Fig. 5:**
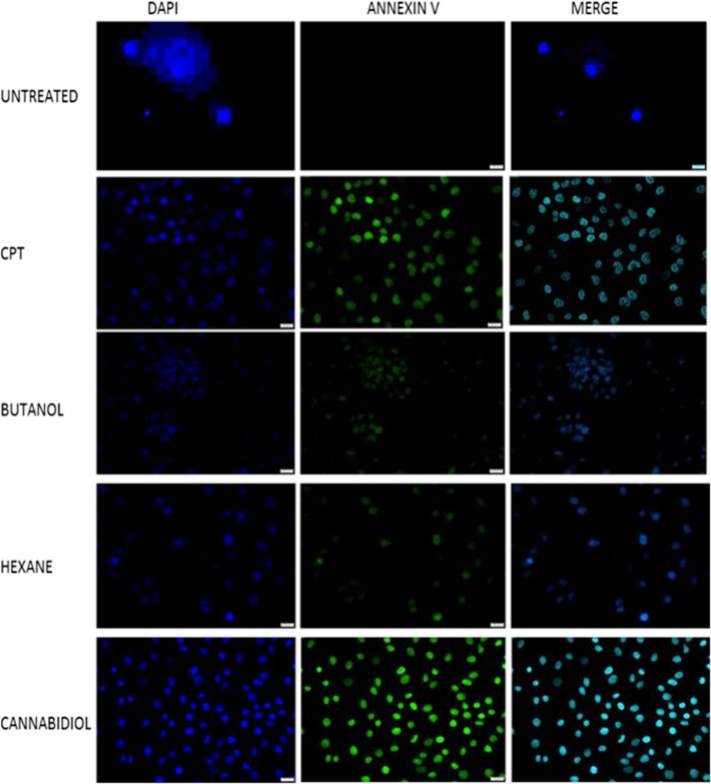
Morphological analysis and assessment of apoptosis in HeLa cells stained with DAPI and Annexin V dye. Cells were incubated with IC_50_ of *Cannabis sativa* extracts for a period of 24 h. Cells were stained with Annexin V and counterstained with DAPI. BX63-fluorescence confocal microscopy was used to visualize the cells

### *Cannabis sativa* extracts and cannabidiol induce apoptosis in cervical cancer cells

Flow cytometry revealed a significant increase in SiHa cells undergoing apoptosis during treatment with butanol (from 2 to 28.5 %) and hexane (from 2 to 17.2 %) as compared to camptothecin with 30.4 %. In HeLa cells, apoptosis was increased to 31.9 % in butanol extract and only 15.3 % in hexane cells (Fig. 3b). A similar events was observed following treatment of ME-180 cells with butanol extract were 44.8 % apoptosis was recorded and 43.2 % in hexane treated cells (Fig. 3c). Cannabidiol was also tested for its ability to induce apoptosis in all three cell lines. The results further confirmed that the type of cell death induced was apoptosis. Figure shows that cannabidiol induced early apoptosis in all three cell lines. Cannabidiol was more effective in inducing apoptosis In comparison to both extracts of Cannabis sativa. In SiHa cells cannabidiol induced 51.3 % apoptosis (Fig. 3d), 43.3 % in HeLa and 28.6 % in ME-180 cell lines (Fig. 3f).

### Effect of *Cannabis sativa* extracts and cannabidiol on the morphology of SiHa and HeLa cells

**Fig. 6:**
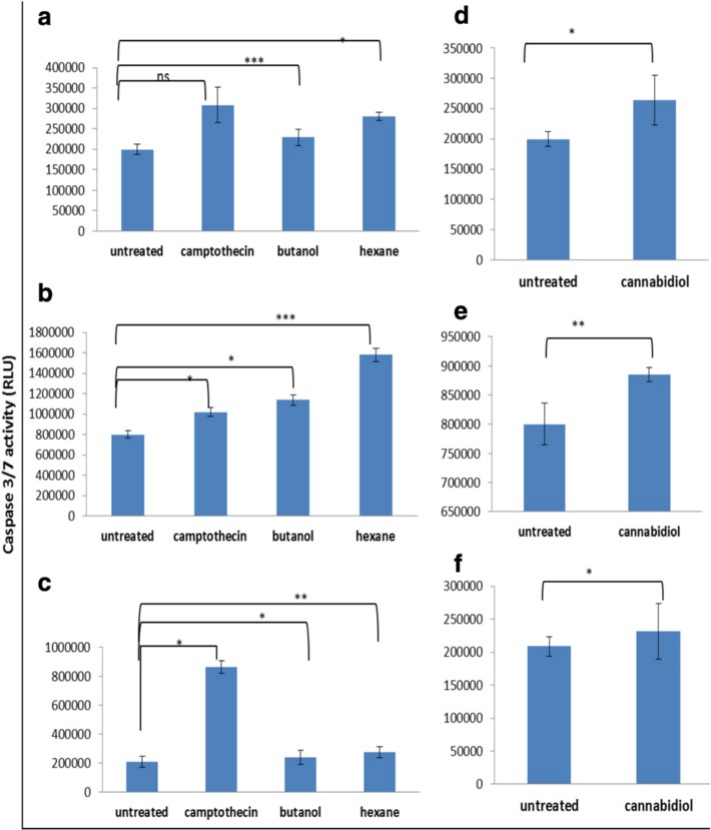
Caspase 3/7 activity after treatment of SiHa, HeLa, and ME-180 cells with IC_50_ of *Cannabis sativa* extract and cannabidiol. Cells were treated with IC_50_ of *Cannabis sativa* and cannabidiol extracts for a period of 24 h. Caspase 3/7 reagent was added to the treated cells for 1 h. Luminescence–was measured using GLOMAX instrument in RLU. Data represented as mean ± standard deviation with ***p < 0.001, **p < 0.01, and *p < 0.05 representing the level of significance in comparison to the untreated

To characterise the cell death type following treatment with our test compounds, cell were stained with DAPI and Annixin V to show if apoptosis was taking place. Treatment of SiHa and HeLa cells with IC_50_ of both butanol and hexane extracts confirmed the type of cell death as apoptosis since they picked a green colour from Annexin V that bind on phosphotidyl molecules that appear in early stages of apoptosis. Similar results were also observed in cannabidiol treated cells. Another feature that is a representative of cell death is the change in morphology. Morphological appearance of live cells displayed a round blue nuclei following staining with DAPI. Exposure of SiHa and HeLa cells to IC_50_ of *Cannabis sativa* extracts caused a change in morphology coupled with an uptake of annexin V. Loss of shape, nuclear fragmentation, reduction in cell size and blebbing of the cell membrane were among the observed morphological features implicated to be associated with apoptosis (Fig. 6).

**Fig. 7:**
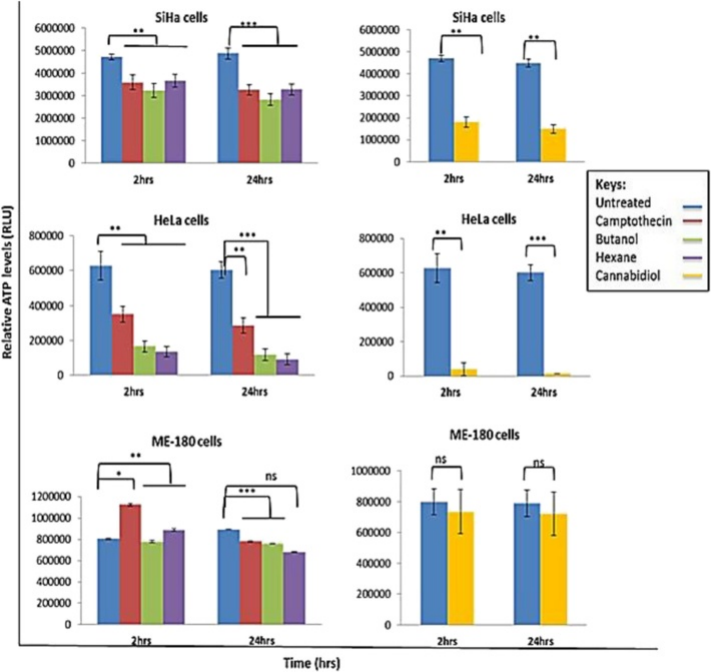
Bar graphs representing changes in the ATP levels following treatment of cervical cancer cells with *Cannabis sativa* and cannabidiol. Cells were treated with IC_50_ of both *Cannabis sativa* extracts and cannabidiol for a period of 2–24 h. Untreated and camptothecin were included as controls for comparative purposes. The level of significance was determined using Students *t*-Test with ***p < 0.001, **p < 0.01, *p < 0.05, and ^ns^ *p* > 0.05 in comparison to the untreated

### Effect of *Cannabis sativa* extracts and cannabidiol on the ATP levels

Since Adenosine 5’-triphosphate acts as a biomarker for cell proliferation and cell death, an ATP assay was conducted. This was done in order to determine whether *Cannabis sativa* and cannabidiol deplete ATP levels in cervical cancer cells. SiHa, HeLa, and ME-180 cells were treated at different time points, between 2 and 24 h. ATP levels were first detected after 2 h. In general, ATP depletion was cell type dependent. In HeLa cells treated with the crude extracts from butanol and hexane, ATP was significantly reduced by 74 % (from 627621 to 164208 RLU) and 78 % (from 627621 to 133693 RLU) respectively. While with SiHa there was reduction of 31 % (from 4719589 to 3221245 RLU) and 22.5 % (4719589 to 3655730 RLU) respectively (figure). Whereas in ME-180 there was no change between treatments and untreated. Similar results were observed in cannabidiol treated cells. At 2 h, treatment with IC_50_ led to a reduction in ATP levels by ~ 61 % (from 4704419 to 1802508 RLU), 93 % (from 627621 to 40371 RLU), and 8 % (from 798688 to 734039 RLU) in SiHa, HeLa, and ME-180 cells respectively (Figure). A prolonged incubation period (24 h) of cells with IC_50_ led to a further decrease in the ATP levels by ~66 % (from 4486150 to 1497648 RLU), 97 % (from 601694 to 13426 RLU), and 8.5 % (from 790757 to 723039 RLU) in SiHa, HeLa, and ME-180 cells respectively. This could mean that cannabidiol depletes ATP levels more than *Cannabis sativa* extracts and might be the main compound responsible for cell death in cancer cells treated with *Cannabis sativa*.

### Effect of *Cannabis sativa* and cannabidiol on caspase 3/7 activity of SiHa, HeLa, and ME-180 cells

**Fig. 8:**
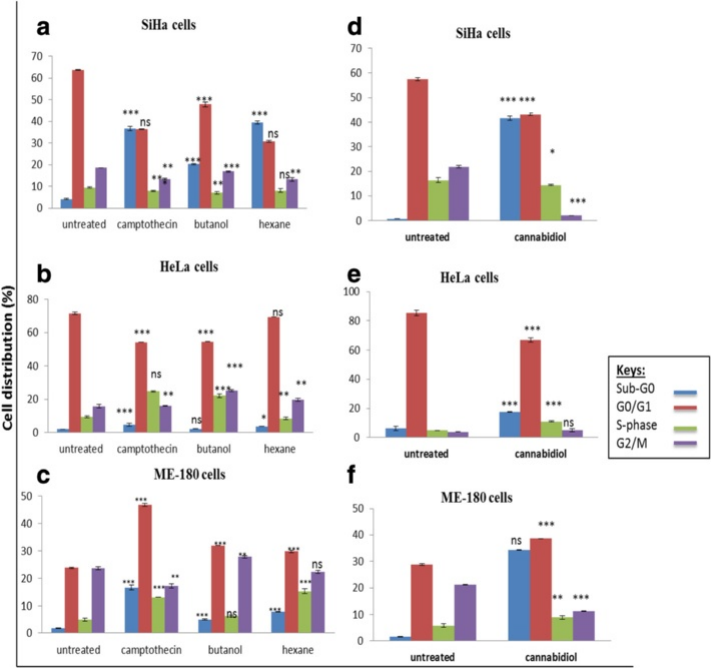
Representative bar graph of the cervical cancer cell cycle before and after treatment with *Cannabis sativa* extracts and cannabidiol. Cells were harvested and treated with camptothecin and the IC_50_ concentrations of *Cannabis sativa* extracts and cannabidiol. Bar graph **a** and **d** represents SiHa cells, **b** and **e** represents HeLa cells, **c** and **f** represents ME-180 cells. Data represented as mean ± standard deviation with ***p < 0.001, **p < 0.01, *p < 0.05, and ^ns^ *p* > 0.05 representing the level of significance in comparison to the untreated

As shown in Fig. 8a, b and c, we observed an increase in caspase 3/7 activity all three cell lines following treatment with 0.3 μM of camptothecin. Similar results were observed in crude extract treated cells by 25 % (SiHa) and 40 % (HeLa) in butanol extract and 50 % (SiHa) and 100 % (HeLa) in hexane treated. There was no significant change in ME-180 cells (Figure). When cells were treated with cannabidiol, caspase 3/7 activity increased in all three cell lines. SiHa cells so an increase from 200000 RLU to 2500000 while HeLa increased to 900000 from 800000 RLU. ME-180 was fairly increased also to 230000 from 200000 RLU all increase were significant and in line with other increase in apoptosis as shown in Annexin V.

### Effect of *Cannabis sativa* extracts and cannabidiol on cell cycle progression

**Fig. 9:**
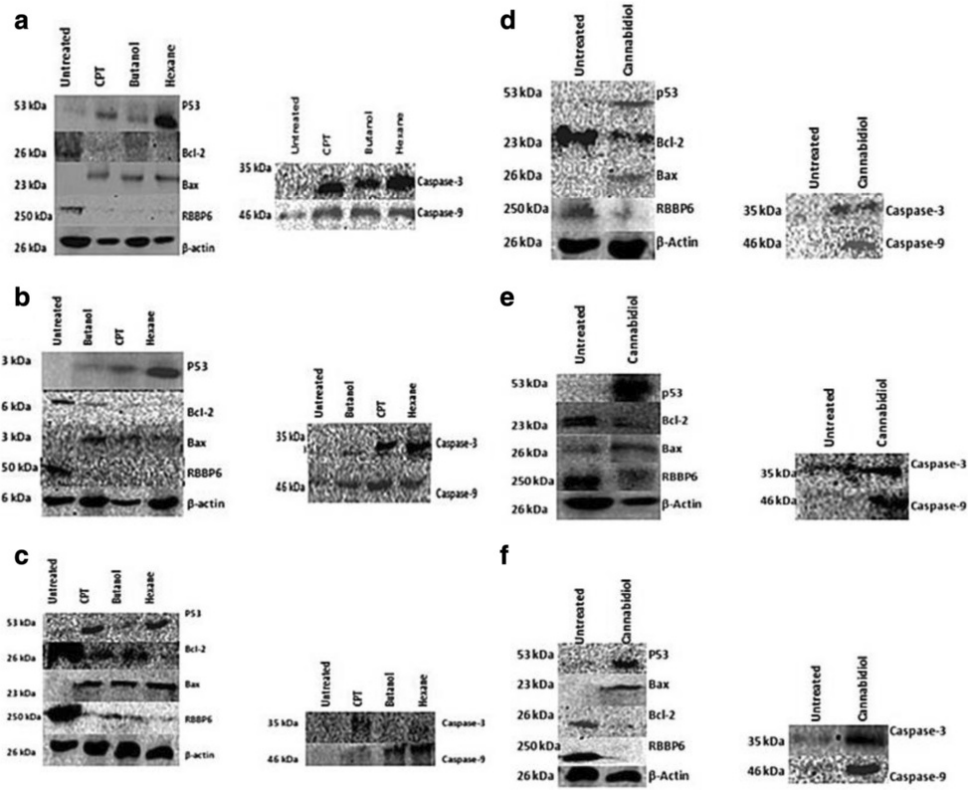
Western blot analysis of the protein expression before and after 24 h treatment with IC_50_ of *Cannabis sativa* extracts and cannabidiol. SiHa (**a** and **d**), HeLa (**b** and **e**), and ME-180 (**c** and **f**) cells were treated for a period of 24 h and protein lysates were separated using SDS-PAGE gel. Untreated protein was used as a control. Antibodies against pro-apoptotic proteins (p53 and Bax) and anti-apoptotic proteins (Bcl-2 and RBBP6), Initiator caspase-9 and effecter caspase-3 were included to elucidate apoptosis induction

We further assessed the effects of *Cannabis sativa* extracts and cannabidiol on cell cycle progression using flow cytometry. Flow cytometry showed that in the presence of *Cannabis sativa* crude extracts and camptothecin, SiHa cells exhibited a significant increase (*p* < 0.001) in sub-G_0_ population with a decrease in G_0_/G_1_, S, and G_2_/M phase. In butanol, sub-G_0_ phase was increased from 4.2 to 20.1 %, while decreasing the G_0_/G_1_ (from 64.0 to 48.7 %), S-phase (from 9.3 to 6.5 %), and G_2_/M (from 18.5 to 17 %) in SiHa population (Fig. 9a) while in hexane treated sub-G_0_ phase was at 39.1 % compared to 4.2 % in untreated, with a decrease in G_0_/G_1_ (from 64.0 to 30.4 %), S (from 9.3 to 6.5 %), and G_2_/M (from 18.5 to 13.8 %) population (Fig. 9a). In HeLa cells, butanol extracts reduced G_0_/G_1_ to 54.9 % while the S-phase and G_2_/M significantly increased to 18.4 and 25.7 % while with hexane there was increase in the G_2_/M phase (20.3 %) and a decrease in the S-phase (8.1 %). In ME-180 there was insignificant increase in all cell cycle stages. Each cell line responded differently to cannabidiol treatment. Almost 42.2 % of SiHa cells were observed in the sub-G0 (*p* < 0.001) while there was reduction in cells in the G0/G1 phase, from 57.9 to 42.8 % (Fig. 9d). A similar trend was observed in HeLa cells but much lower sub-G_0_ (from 5.1 to 17.4 %) and S phase (from 4.8 to 11.2 %) (Fig. 9e). A similar event was observed during treatment of ME-180 cells. Cannabidiol significantly increased sub-G0 in ME-180 cells to 34.3 % (Fig. 9f). From this data, we can conclude that cannabidiol induced cell death without cell cycle arrest.

**Fig. 10:**
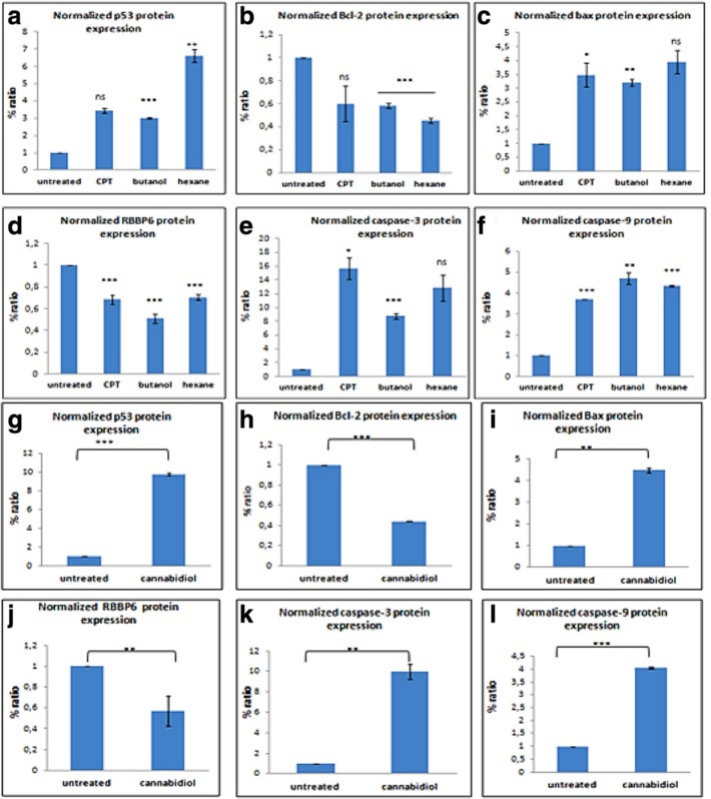
A densitometry analysis SiHa protein was performed using ImageJ quantification software to measure the relative band intensity. CPT represents camptothecin. Data represented as mean ± standard deviation with ***p < 0.001, **p < 0.01 and ^ns^ *p* > 0.05 representing the level of significance in comparison to the untreated represent the western blot analysis of SiHa and HeLa cells. The genes analyzed are p53 and RBBP6 including caspases. Equal amount of protein (conc) was loaded in each well. Note that the darker the bands increased expression of the gene

**Fig. 11:**
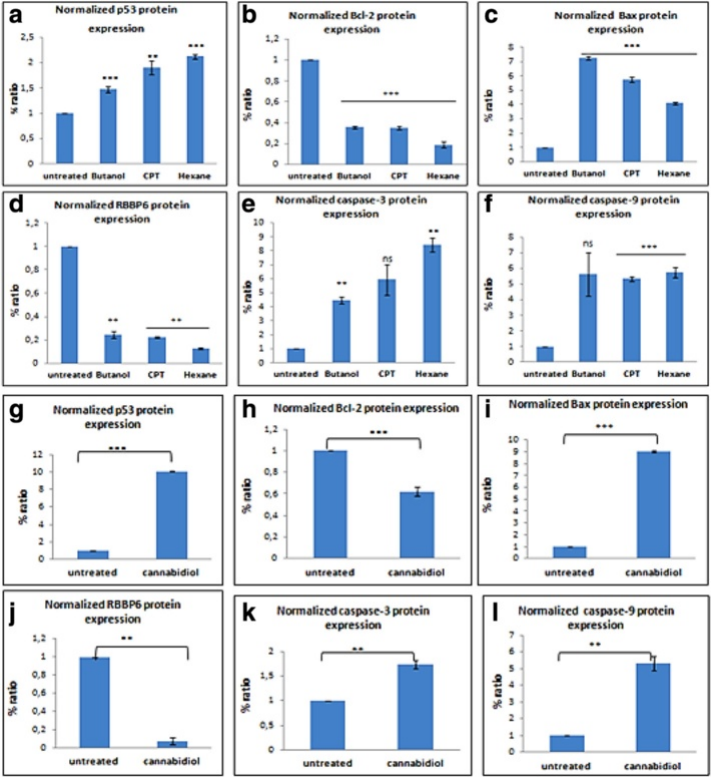
A densitometry analysis HeLa protein was performed using ImageJ quantification software to measure the relative band intensity. CPT represents camptothecin. Data represented as mean ± standard deviation with ***p < 0.001, **p < 0.01 and ^ns^ *p* > 0.05 representing the level of significance in comparison to the untreated

**Fig. 12:**
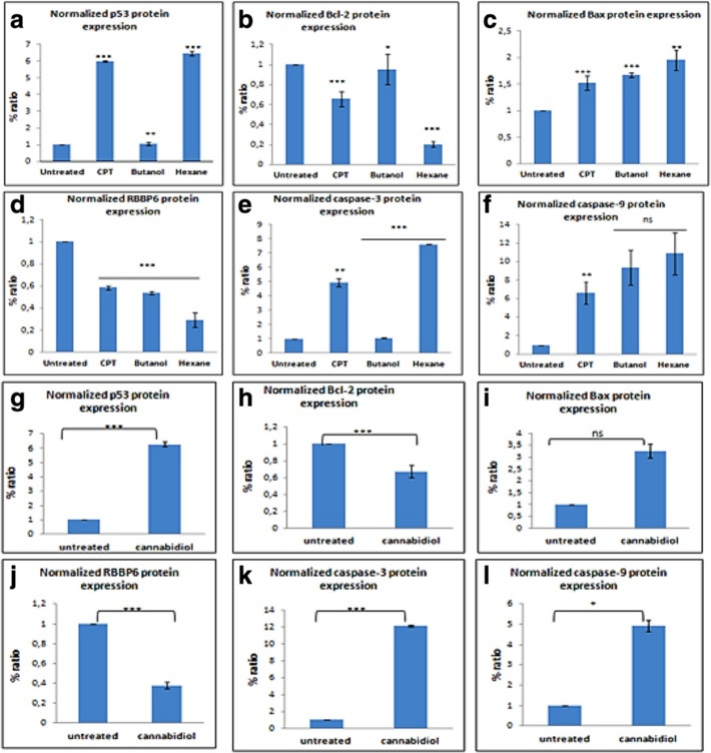
A densitometry analysis ME-180 protein was performed using ImageJ quantification software to measure the relative band intensity. CPT represents camptothecin. Data represented as mean ± standard deviation with ***p < 0.001, **p < 0.01 and ^ns^ *p* > 0.05 representing the level of significance in comparison to the untreated

### Effect of *Cannabis sativa* extracts and cannabidiol on the expression of upstream and downstream target proteins

From the apoptosis experiments conducted, it is clear that the mode of cell death induced by cannabidiol and extract of Cannabis Savita was that of apoptosis. However, we needed to confirm whether the type of apoptosis induced is it p53 dependent or independent as it is well known that p53 is mutated in many cancers. Protein expression of RBBP6, Bcl-2, Bax and p53were performed and results recorded. In butanol extract p53 was significantly increased in SiHa and HeLa cells while remaining unchanged in ME-180. Similar results were observed in hexane treated cells. In all cell lines the level of p53 negative regulator in cancer development was reduced by all treatment.

Following treatment of cervical cancer cells, Bax protein was up-modulated and Bcl-2 was down-modulated. Western blot analysis revealed that cannabidiol effectively caused an increase in the expression of pro-apoptosis proteins, p53 and Bax, while simultaneously decreasing the anti-apoptosis proteins, RBBP6 and Bcl-2 in all three cervical cancer cell lines (SiHa, HeLa, and ME-180 cells). Caspases play an effective role in the execution of apoptosis, an effector caspase-9 and executor capsase-3 were included in our western blot to check if they played a role in inducing apoptosis. In all *Cannabis sativa* extracts, caspase-3 and caspase-9 were upregulated in all cell lines. Similar results were also observed in cannabidiol treated cells with upregulation of both caspase-3 and -9.

## Discussion

Cervical cancer remains a burden for women of Sub-Saharan Africa. Half a million new cases of cervical cancer and a quarter of a million deaths are reported annually due to lack of effective treatment [12]. Currently, the recommended therapeutic regimens include chemotherapy, radiation therapy, and surgery. However, they present several limitations including side effects or ineffectiveness [2]. Therefore, it is important to search for new novel therapeutic agents that are naturally synthesized and cheaper, but still remain effective. Medicinal plants have been used for decades for health benefits and to treat several different diseases [22]. In South Africa, over 80 % of the population are still dependent on medicinal plants to maintain mental and physical health [27]. However, some of the medicinal plants used by these individuals are not known to be effective and their safety is still unclear. It is therefore important to scientifically evaluate and validate their efficacy and safety. In the present study, cervical cancer cell lines (SiHa, HeLa, and ME-180) were exposed to different concentrations of *Cannabis sativa* extracts and that of its compound, cannabidiol, with the aim of investigating their anti-proliferative activity.

We first determined whether *Cannabis sativa* extracts and cannabidiol possess anti-proliferative effects using MTT assay. MTT assay determines IC_50_, which represents the half maximal concentration that induces 50 % cell death. *Cannabis sativa* extracts were able to reduce cell viability and increase cell death in SiHa, HeLa, and ME-180 cells. These results correlate with the findings obtained by [23], whereby they reported reduced cell proliferation in colorectal cancer cell lines following treatment with *Cannabis sativa*. According to [7, 24, 25] *Cannabis sativa* extracts rich in cannabidiol were able to induce cell death in prostate cancer cell lines LNCaP, DU145, and PC3 at low doses (20–70 μg/ml). It was suggested that cannabidiol might be responsible for the reported activities. Therefore, in this study, cannabidiol was included as a reference standard in order to determine whether the reported pharmacological activities displayed by *Cannabis sativa* extracts might have been due to the presence of this compound. For positive extract inhibitory activity, Camptothecin was included as a positive control. Camptothecin functions as an inhibitor of a topoisomerase I enzyme that regulates winding of DNA strands [19, 20]. This in turn causes DNA strands to break in the S-phase of the cell cycle [20]. A study conducted by [19], exhibited the ability of camptothecin to be cytotoxic against MCF-7 breast cancer cell line and also induce apoptosis as a mode of cell death at 0.25 μM. We also observed a similar cytotoxic pattern, whereby camptothecin induced cell death in HeLa, SiHa, and ME-180 cells, however, at a much higher concentration.

xCELLigence continuously monitors cell growth, adhesion, and morphology in real-time in the presence of a toxic substance. Upon treatment of SiHa and HeLa cells with IC_50_ of butanol extract, we noted that there was little to no inhibitory effect observed on cell growth. The growth curve continued in its exponential growth in all cells including the treated, untreated and 0.1 % DM’SO. However, at a similar IC_50_ of 100 μg/ml, a reduction in cell viability was observed following treatment of HeLa cells with hexane extract. On the other hand, ME-180 cells responded after a period of 2 h following treatment with the IC_50_ of butanol and hexane extract. In comparison to butanol and hexane extracts, cannabidiol reduced the cell index of ME-180 cells after 2 h of treatment, signalling growth inhibition. Differences in the findings could be attributable to the fact that both methods have different principles and mechanism of action. MTT assay is an end-point method that is based on the reduction of tetrazolium salt into formazan crystals by mitochondrial succinate dehydrogenase enzyme. Mitochondrial succinate dehydrogenase is only active in live cells with an intact metabolism [8, 13]. Induction of cell death by *Cannabis sativa* crude extracts decreases the activity of the enzyme following treatment of HeLa, SiHa, and ME-180 cervical cancer cell lines. On the other hand, xCELLigence system is a continuous method that relies on the use of E-plates engraved with gold microelectrodes at the bottom of the plate. The xCELLigence system is based on the changes in impedance influenced by cell number, size and attachment [13]. Therefore, we concluded that it was possible that dead cells might have been attached at the bottom of the E-plate after treatment.

Cell death can be characterized by a decrease in the energy levels as a result of dysfunction of the mitochondria [8]. Therefore, to evaluate the effect of treatment on the energy content of the cells, we conducted mitochondrial assay. We only used IC_50_ as indicated by MTT assay only. ATP acts as determinant of both cell death and cell proliferation [15]. Exposure of SiHa, HeLa, and ME-180 cells to the IC_50_ of *Cannabis sativa* extracts caused a reduction in the ATP levels. Treatment of cells with cannabidiol either slightly or severely depleted the ATP levels. According to [16], a reduction of the ATP levels compromises the status of cell and often leads to cell death either by apoptosis or necrosis, while an increase is indicative of cell proliferation. Therefore, we concluded that the reduction of ATP might have been as a result of cell death induction since the cells ATP production recovered.

Following confirmation that *Cannabis sativa* and cannabidiol have anti-proliferative activity, we had to verify whether both treatments have the ability to induce cell cycle arrest in all three cell lines. This method uses a PI stain and flow cytometry to measure the relative amount of DNA present in the cells. In this study, propidium iodide (PI) was used to stain cells. Propidium iodide can only intercalate into the DNA of fixed and permeabilized cells with a compromised plasma membrane or cells in the late stage of apoptosis. Viable cells with an intact plasma membrane cannot uptake the dye. The intensity of stained cells correlates with the amount of DNA within the cells. HeLa, SiHa, and ME-180 cervical cancer cells were stained with PI and analysed using flow cytometry. Treatment of SiHa cells with butanol and hexane extracts led to the accumulation of cells in the cell death phase (sub-G_0_ phase), without cell cycle arrest. When compared to the S-phase and G_2_/M phase of untreated cells, exposure of HeLa cells to *Cannabis sativa* butanol extract resulted in the accumulation of cells in the S-phase of the cell cycle and slight cell death induction. And thus, according to [3], signals DNA synthesis and cell cycle proliferation. A decrease in the S-phase and an increase in the G_2_/M phase of HeLa cells following treatment with hexane extract, suggests a blockage of mitosis and an induction of cell cycle arrest. Interesting to note was that, treatment of ME-180 cells with both extracts led to an increase of cells coupled by an increase in the S-phase population which favours replication and duplication of DNA. This was not the case following treatment of cells with cannabidiol. Cannabidiol resulted in the accumulation of cells in the cell death phase of the cell cycle. SiHa, and HeLa, and ME-180 cells were committed to the cell death phase. In summary, *Cannabis sativa* induces cell death with or without cell cycle arrest while cannabidiol induces cell death without cell cycle arrest.

Apoptosis plays a major role in determining cell survival. Annexin V/FITC and PI were used to stain the cells to be able to distinguish between viable, apoptotic and necrotic cells. Annexin V/ FITC can only bind to phosphatidylserine residues exposed on the surface of the cell membrane while PI intercalates into the nucleus and binds to the fragmented DNA. Viable cells cannot uptake both dyes due to the presence of an intact cell membrane. Since treatment caused the accumulation of cells in the sub-G_0_ phase, also known as the cell death phase, and the severe depletion of ATP levels by cannabidiol, we further conducted an apoptosis assay. Treatment of all three cell lines with camptothecin, IC_50_ of *Cannabis sativa* and cannabidiol exhibited the type of induced cell death as apoptosis. Sharma et al. [25] also showed a similar pattern of cell death, whereby treatment of a prostate cancer cell lines with *Cannabis sativa* resulted in the induction of apoptosis.

Apoptosis is characterized by morphological changes and biochemical features which include condensation of chromatin, convolution of nuclear and cellular outlines, nuclear fragmentation, formation of apoptotic blebs within the plasma membrane, cell shrinkage due to the leakage of organelles in the cytoplasm as well as the presence of green stained cells at either late or early apoptosis [5, 17, 28]. Annexin V/FITC and DAPI were used to visualize the cells under a fluorescence confocal microscopy. According to [18], an uptake of Annexin V/FITC suggests the induction of apoptosis, since it can only bind to externalized PS residues. This also proves that during cell growth analysis, SiHa and HeLa cells were undergoing cell death while still attached to the surface of the flask.

Apoptosis is known to occur via two pathways, the death receptor pathway and the mitochondrial pathway [30]. *Cannabis sativa* isolates including cannabidiol have been implicated in apoptosis induction via the death receptor pathway, by binding to Fas receptor or through an activated of Bax triggered by the synthesis of ceramide in the cells [4]. However, not much has been reported on the induction of apoptosis via activation of p53 by *Cannabis sativa*. Our focus in this study was also to identify downstream molecular effect of extracts. One such important gene is p53 which acts as a transcription factor for a number of target genes [29]. Under normal conditions, p53 levels are maintained through constant degradation MDM2 and its monomers [29]. RBBP6 is one of the monomers that helps degrade p53, due the presence of Ring finger domain that promotes the interaction of both proteins [14]. In response to stress stimuli such as DNA damage, hypoxia, UV light, and radiation light, p53 becomes activated and causes MDM2 expression to decrease [10]. Mutation of p53, implicated to be associated with 50 % of all human cancers, promote the tumorigenesis. Bax and Bcl-2 form part of the proteins that regulate apoptosis via the mitochondria [21]. Following activation, p53 translocates into the cytosol and triggers the oligomerization of Bcl-2 with BAD, resulting in the inhibition of Bcl-2 activity [17]. This in turn allows Bax protein to be translocated to the mitochondria and participate in the release of cytochrome c through poration of the outer mitochondrial membrane [9, 17]. An imbalance between Bax and Bcl-2 has been linked to the development and progression of tumours through the resistance of apoptosis [17]. It is therefore crucial to design drugs that would effectively target these genes involved in the execution of apoptosis via the mitochondrial pathway. Camptothecin, hexane extract, and cannabidiol effectively up-modulated the expression of p53 in all three cell lines, leading to a decrease in RBBP6 protein expression. Apart from SiHa and HeLa, butanol extract failed to up-modulate p53 in ME-180 cells. Interesting to note is that butanol extract reduced the expression of RBBP6 protein in ME-180 cells. The mechanism behind failure of butanol to up-modulate p53 while down-modulating RBBP6 is unclear. However, we came to a conclusion that butanol induces apoptosis independently of p53. We further demonstrated that *Cannabis sativa* extracts, cannabidiol, and camptothecin were able to down-modulate the expression of Bcl-2 protein and up-modulate Bax expression.

Caspases play an effective role in the execution of apoptosis either through the extrinsic or intrinsic pathway [9]. In this study, we wanted to validate whether caspase-9 and caspase-3 were involved in the initiation and execution of apoptosis. We demonstrated the ability of *Cannabis sativa* to initiate apoptosis by activating caspase-9. However, execution of apoptosis was either with or without the presence of capsase-3, depending on each cell line. Western blot revealed that *Cannabis sativa* hexane extract induced apoptosis via the activation of caspase-9 and caspase-3 when compared to untreated cells in all three cell lines. Similar results were obtained during treatment of all three cell lines with camptothecin. This was not the case with butanol. Butanol extracts up-modulated caspase-9 and caspase-3 in SiHa and HeLa cells only. Caspase-3 was not up-modulated in ME-180 cells. Caspase 3/7 activity assay revealed the up-modulation of caspase 3/7 following treatment of cervical cancer cells. However on the basis of the Western blot results, wherein butanol extract failed to up-modulate caspase-3, we can conclude that caspase-7 was responsible for the reported activity. Cannabidiol effectively up-modulated caspase-9 and caspase-3 in all three cell lines, when compared to the untreated and *Cannabis sativa* extract. From the results we can conclude that, apoptosis induction was caspase dependent.

## Conclusions

The aim of this study was to evaluate for the anti-growth effects of *Cannabis sativa* extracts and to also determine the mode of cell death following treatment. The activity of *Cannabis sativa* extracts was compared to that of cannabidiol, in order to verify whether the reported results were due to the presence of the compound. The study showed that the activity of one of the extracts might have been due to the presence of cannabidiol. It further demonstrated the ability of *Cannabis sativa* to induce apoptosis with or without cell cycle arrest and via mitochondrial pathway. More research needs to be done elucidating the mechanism between the active ingredients and molecular targets involved in the regulation of the cell cycle.

## Abbreviations

Bad: Bcl-2-associated death promoter
Bak-1: Bcl2-antagonist/killer 1
Bax: Bcl2-associated X protein
Bcl-2: B-cell lymphoma 2
BH: Bcl-2 homology domain
Bid: BH3 interacting-domain
Bik: Bcl-2-interacting killer
DMEM: Dulbecco’s modified eagle’s medium
DMSO: Dimethyl sulfoxide
FITC: Fluorescein isothiocyanate
P53: protein 53
HPLC: High Perfomance Liquid Chromatography
RBBP6: Retinoblastoma binding protein 6
